# Loss of Lysyl Oxidase-like 3 Attenuates Embryonic Lung Development in Mice

**DOI:** 10.1038/srep33856

**Published:** 2016-09-20

**Authors:** Jian Zhang, Ziyi Liu, Tingting Zhang, Zhuchun Lin, Zhenzu Li, Aizhen Zhang, Xiaoyang Sun, Jiangang Gao

**Affiliations:** 1 School of Life Science and Key Laboratory of the Ministry of Education for Experimental Teratology, Shandong University, Jinan 250100, China; 2 Jinan First People’s Hospital, Jinan 250011, China

## Abstract

Lysyl oxidase-like 3 (LOXL3), a human disease gene candidate, is a member of the lysyl oxidase (LOX) family and is indispensable for mouse palatogenesis and vertebral column development. Our previous study showed that the loss of LOXL3 resulted in a severe cleft palate and spinal deformity. In this study, we investigated a possible role for LOXL3 in mouse embryonic lung development. LOXL3-deficient mice displayed reduced lung volumes and weights, diminished saccular spaces, and deformed and smaller thoracic cavities. Excess elastic fibres were detected in LOXL3-deficient lungs, which might be related to the increased LOXL4 expression. Increased transforming growth factor β1 (TGFβ1) expression might be involved in the up-regulation of LOXL4 in LOXL3-deficient lungs. We concluded that the loss of LOXL3 attenuates mouse embryonic lung development.

Lysyl oxidases are extracellular copper-dependent amine oxidases that initiate the crosslinking of collagens and elastin^1^. Lysyl oxidase (LOX) family members exhibit similar, but not identical, expression patterns^2^, suggesting that LOX family members may have unique functions in different tissues. LOX activates the covalent cross-links of several fibrillar collagen types and the formation of desmosine and isodesmosine cross-links in elastin. LOX knockout mice exhibit perinatal lethality, cardiovascular instability with ruptured arterial aneurysms, and diaphragmatic rupture^2^,^3^. However, the function of LOXL1 differs from that of LOX, and LOXL1-deficient mice display pelvic organ prolapse, enlarged airspaces in the lungs, loose skin, and vascular abnormalities with concomitant tropoelastin accumulation^4^.

LOX family members, including LOXL3, are characterized by a highly conserved C-terminal amino acid sequence that includes a copper binding site, residues required for the formation of the carbonyl co-factor, and a cytokine receptor-like domain^5^,^6^. In humans, LOXL3 and its variant LOXL3-sv1 have high amine oxidase activities on several collagen types, suggesting that LOXL3 has a possible functional role in bone development or maintenance^7^. Recently, a missense variant (exon12:c.2027G > A) in the human *LOXL3* gene was identified in a family with autosomal recessive Stickler syndrome^8^, which is caused by mutations in *COL2A1*, *COL11A1*, *COL11A2*, *COL9A1* and *COL9A2*. This syndrome is one of the most common connective tissue disorders and is characterized by ocular, skeletal, orofacial and auditory defects^9^,^10^,^11^. Thus, *LOXL3* was identified as a candidate human disease gene in the LOX family. The lack of Loxl3b, an ortholog of mammalian LOXL3, also caused craniofacial defects in zebrafish^12^. In our previous study, we generated LOXL3 knockout mice. These mice exhibited perinatal lethality, impaired development of the palate shelves, and abnormalities in the cartilage primordia of the thoracic vertebrae^13^. We showed that the decreased collagen cross-links in the palate and spine induced by the lack of LOXL3 resulted in cleft palates and spinal deformities. In addition, we also observed a reduction in the saccular space in the lungs of LOXL3-deficient mice at embryonic day 18.5 (E18.5)^13^. In the current study, we investigated the effect of LOXL3 on mouse lung development, and examined the lung phenotypes of LOXL3-deficient mice at different embryonic stages. We discovered aberrant, immature lung development in LOXL3-deficient mice at different embryonic periods, suggesting that the loss of LOXL3 attenuated mouse embryonic lung development.

## Results

### Lung defects in the LOXL3-deficient mice at different embryonic developmental periods

**Figure 1:**
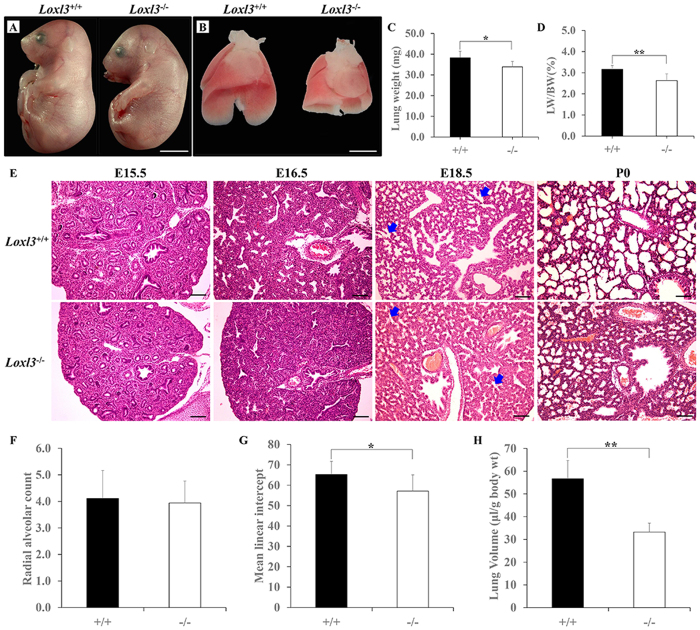
Lung defects in LOXL3 knockout mice. (**A**) *Loxl3*^−/−^ mice exhibited spinal abnormalities at E18.5. Bar: 5 mm. (**B**) The *Loxl3*^−/−^ mouse lungs were smaller in size than the *Loxl3*^+/+^ mouse lungs at E18.5. Bar: 2 mm. (**C,D**) Lung weights and the LW/BW ratios of the *Loxl3*^−/−^ mice were significantly decreased compared with the *Loxl3*^+/+^ mice at E18.5. *P < 0.05. **P < 0.01. (**E**) No significant differences appeared in the lung structures between *Loxl3*^−/−^ and *Loxl3*^+/+^ mice until E15.5. The terminal lung tubules (acini) of the *Loxl3*^−/−^ mice did not dilate at E16.5. A significant reduction in the distal saccular spaces in *Loxl3*^−/−^ lungs was noted from E18.5 onwards. Blue arrows: distal saccules. Bar: 100 μm. (**F**) Normal RACs were observed in the *Loxl3*^−/−^ mouse lungs at E18.5. (**G**) Decreased MLIs were observed in the *Loxl3*^−/−^ mouse lungs at E18.5. *P < 0.05. (**H**) The lung volumes of the *Loxl3*^−/−^ mice were also significantly decreased at E18.5. **P < 0.01. For all bar graphs, n = 5 in each group.

Newborn LOXL3-deficient (*Loxl3*^−/−^) mice died shortly after birth. The *Loxl3*^−/−^ mice displayed spinal deformities (Fig. 1A) and lung defects (Fig. 1B) at E18.5. The lung weights (LW) and body weights (BW) of the E18.5 mice were measured. The LW were significantly lighter, and the LW/BW ratio was significantly decreased in the *Loxl3*^−/−^ mice compared with the wild-type (*Loxl3*^+/+^) mice (Fig. 1C,D). Paraffin sectioning and H&E staining were performed to investigate mouse embryonic lung development in detail. We did not observe significant differences between the lung structure of the *Loxl3*^+/+^ mice and that of the *Loxl3*^−/−^ mice until E15.5 (Fig. 1E). A histological analysis of the lungs from the *Loxl3*^+/+^ mice showed the sequential process of normal airspace morphogenesis (Fig. 1E). However, the terminal lung tubules (acini) in the *Loxl3*^−/−^ mice did not expand at E16.5 and the distal saccular spaces in the *Loxl3*^−/−^ lungs were reduced compared with the *Loxl3*^+/+^ lungs from E18.5 onwards (Fig. 1E). At E18.5, the radial alveolar counts (RAC) analysis showed a normal number of air saccules in the *Loxl3*^−/−^ mice (Fig. 1F). The observations of the mean linear intercept (MLI), a reflection of airspace dimension, indicated that LOXL3 deficiency reduced the size of the saccular space (Fig. 1G). Based on body weight, the lung volumes of the *Loxl3*^−/−^ mice were also decreased by 41.3% (Fig. 1H).

### Thorax deformities in the LOXL3-deficient mice

**Figure 2:**
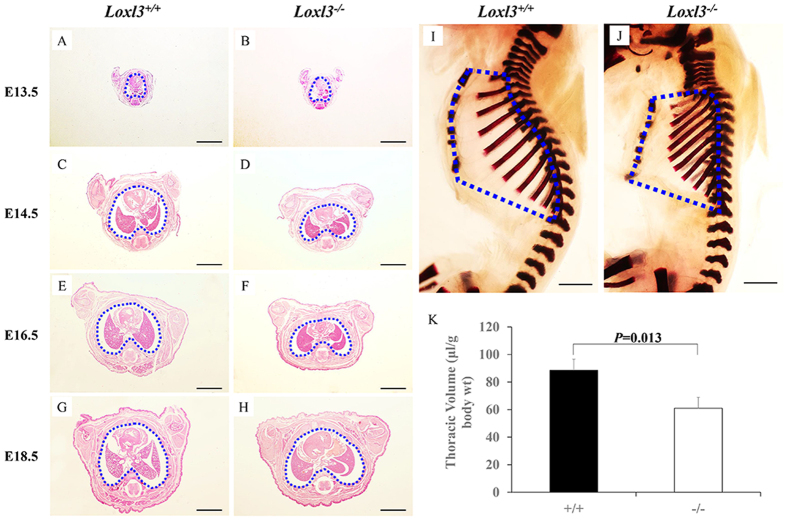
Thoracic defects in the LOXL3-deficient mice. (**A–H**) Transverse view of H&E staining of mouse thorax sections from E13.5 to E18.5. The transverse sectional profiles of the LOXL3-deficient thoraxes tended to be flat from E14.5 to E18.5 compared with the wild-type thoraxes. Bar: 2 mm. (**I,J**) Lateral view of Alizarin Red staining of mouse skeletons at E18.5. The side profiles of the LOXL3-deficient thoraxes also exhibited spine and rib deformities. Bar: 2 mm. (**K**) The thoracic volumes in the *Loxl3*^−/−^ mice were also significantly decreased at E18.5. n = 3 in each group, *P* = 0.013.

The LOXL3-deficient mice displayed spinal deformities. We considered that the spinal deformity might affect the structure of the thorax. Therefore, we examined the thoracic changes in the LOXL3-deficient mice. We did not observe any differences in the transverse thorax sections from the LOXL3-deficient and wild-type mice at E13.5 (Fig. 2A,B). The thorax abnormalities in the LOXL3-deficient mice appeared at E14.5 and worsened over time (Fig. 2C–H). We also examined the bone structure of the thorax and discovered spine and rib deformities in the thoraxes of LOXL3-deficient mice (Fig. 2I,J). Based on body weight, the 31.1% decrease in thoracic volume for the LOXL3-deficient mice compared with the wild-type mice was statistically significant (Fig. 2K).

### LOXL3 expression in mouse lungs

**Figure 3:**
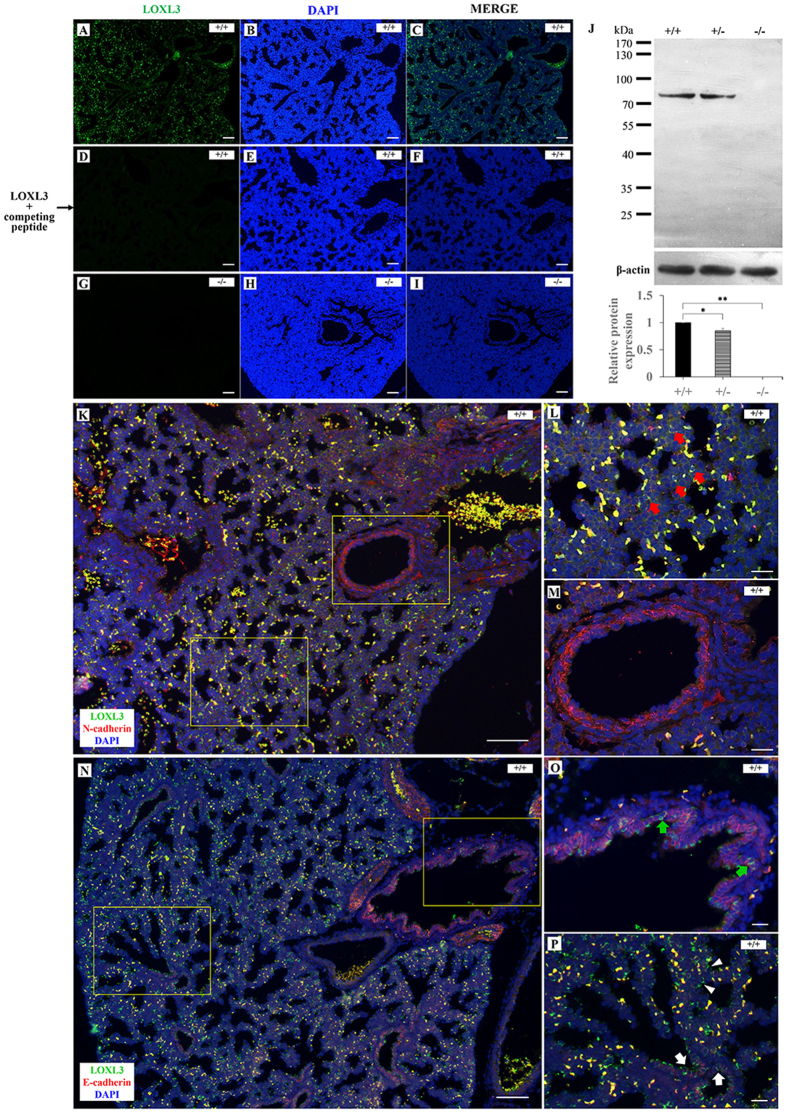
LOXL3 expression in lungs at E18.5. (**A–C**) Immunofluorescence staining indicated that LOXL3 was abundantly expressed in wild-type mouse lungs. (**D–F**) No positive signal was detected by the LOXL3 antibody in wild-type mouse lungs in the presence of the LOXL3 competing peptide. (**G–I**) LOXL3 expression was not detected in LOXL3-deficient mouse lungs. (**J**) Western blots for LOXL3 and β-actin in *Loxl3*^+/+^, *Loxl3*^+/−^and *Loxl3*^−/−^ lungs were analysed at E18.5. *P < 0.05, **P < 0.01. n = 5 in each group. (**K**) The co-staining of LOXL3 (green) and N-cadherin (red, a mesenchymal marker) in wild-type mouse lungs at E18.5. (**L,M**) Enlarged views of Fig. K. (**L**) N-cadherin stained the mesenchyme, and LOXL3 was found to localize primarily in the mesenchyme (red arrows). (**M**) LOXL3 was rarely expressed in the blood vessels of the lung. (**N**) The co-staining of LOXL3 (green) and E-cadherin (red, an epithelial marker) in wild-type mouse lungs at E18.5. (**O,P**) Enlarged views of Fig. N. (**O,P**) LOXL3 was expressed at lower levels at the interface between the epithelial cells of the proximal airways (green arrows), distal airways (white arrows) and respiratory epithelium (white arrowheads). DAPI was used to stain the nucleus. Bars: 100 μm (**A–I,K,N**); 25 μm (**L,M,O,P**).

We examined the expression of the *Loxl3* gene in mouse lungs to investigate the effects of LOXL3 on lung development. Immunofluorescence staining was performed on E18.5 transverse lung sections. LOXL3 was expressed at high levels in wild-type mouse lungs (Fig. 3A–C). Antibody binding was blocked by the addition of excess antigenic peptide to test the specificity of the LOXL3 antibody; we did not detect a positive fluorescence signal with the LOXL3 antibody in wild-type mouse lungs (Fig. 3D–F). Moreover, no expression of the LOXL3 protein was detected in the LOXL3-deficient mouse lung by immunofluorescence staining (Fig. 3G–I). Western blot also confirmed the loss of LOXL3 protein expression in the E18.5 LOXL3 knockout lung (Fig. 3J). The co-staining of LOXL3 and N-cadherin was performed in wild-type mouse lungs at E18.5 (Fig. 3K). We found that the LOXL3 protein was predominantly expressed in the pulmonary mesenchyme (Fig. 3L). LOXL3 expression was rarely detected in the blood vessels of the lung (Fig. 3M). The co-staining of LOXL3 and E-cadherin was also performed in wild-type mouse lungs at E18.5 (Fig. 3N). Some LOXL3 expression was observed on the interface between the epithelial cells of the proximal airways, distal airways and respiratory epithelium (Fig. 3O,P).

### Excessive accumulation of elastic fibres in LOXL3-deficient lungs

**Figure 4:**
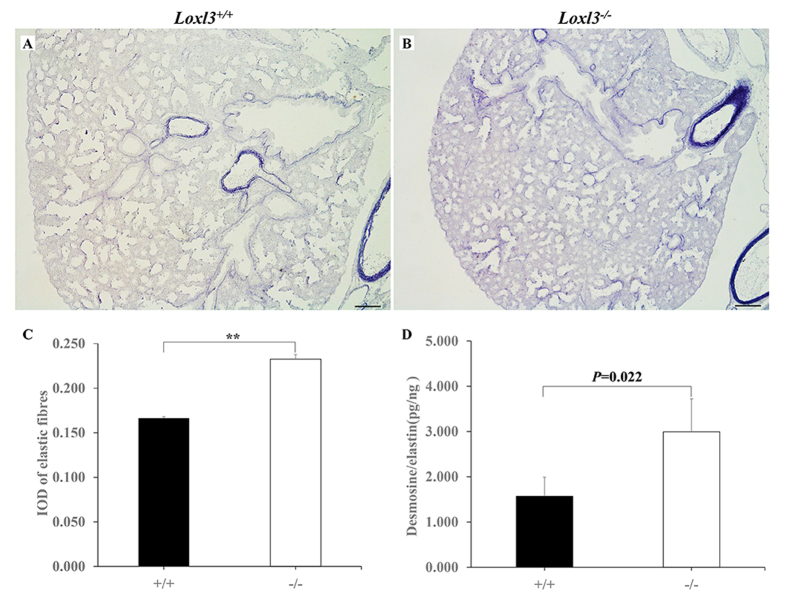
Excessive elastic fibres in the E18.5 LOXL3-deficient lungs. (**A,B**) The distribution of elastic fibres in the *Loxl3*^+/+^ and *Loxl3*^−/−^ lungs was examined by resorcin-fuchsin staining. Bar: 100 μm. (**C**) Mean IOD of the elastic fibres in the *Loxl3*^+/+^ and *Loxl3*^−/−^ lungs. The elastic fibre density in the *Loxl3*^−/−^ mice was significantly increased compared with the *Loxl3*^+/+^ mice. n = 5 in each group, **P < 0.01. (**D**) The desmosine/elastin ratio was significantly increased in the *Loxl3*^−/−^ mice compared with the *Loxl3*^+/+^ mice. n = 6 in each group, *P* = 0.022.

**Figure 5:**
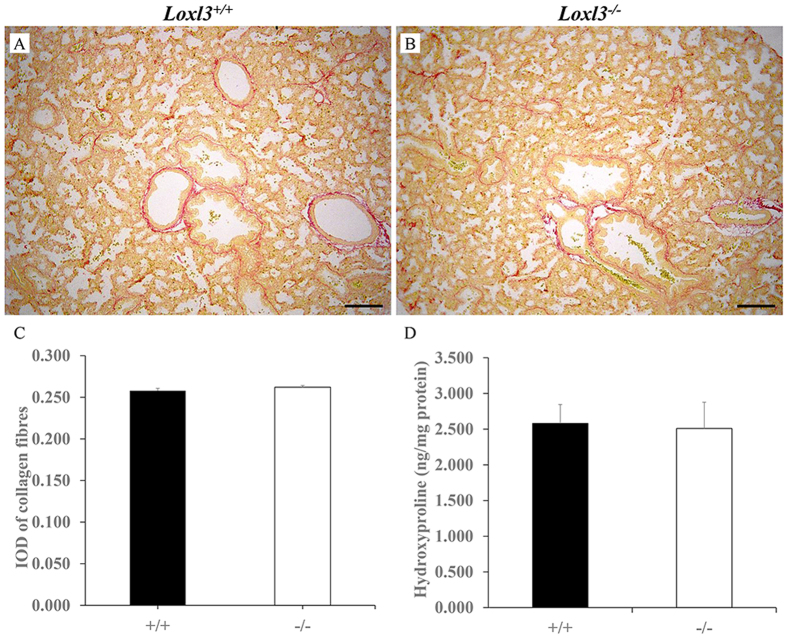
Sirius red staining and hydroxyproline assay in E18.5 lungs. (**A–C**) Sirius red staining showed that there were no differences in the distribution of collagen fibres between the *Loxl3*^−/−^ and *Loxl3*^+/+^ mice. n = 5 in each group. Bar: 100 μm. (**D**) The hydroxyproline concentrations were analysed using a hydroxyproline assay kit. No significant difference was observed between the *Loxl3*^−/−^ and *Loxl3*^+/+^ mice. n = 5 in each group.

LOX family members, including LOXL3, initiate the formation of cross-links in collagen and elastin. Elastic fibres, which stretch and contract during inhalation and exhalation, are major components of the mammalian lung^14^. We investigated the changes in elastic fibres in mouse lungs. Elastic fibres were significantly increased in the *Loxl3*^−/−^ lungs compared with the *Loxl3*^+/+^ lungs at E18.5 (Fig. 4A–C). We also analysed the desmosine and elastin concentrations in the mouse lung by ELISA at E18.5. The desmosine/elastin ratio in *Loxl3*^−/−^ mice was significantly increased compared with the *Loxl3*^+/+^ mice (Fig. 4D). In addition to the elastic fibres, we also examined the distribution of collagen fibres in mouse lungs. Using Sirius red staining, no significant difference was observed in collagen distribution between the *Loxl3*^−/−^ and *Loxl3*^+/+^ mice at E18.5 (Fig. 5A–C). A hydroxyproline assay also showed that the hydroxyproline content of the *Loxl3*^−/−^ mice was similar to the *Loxl3*^+/+^ mice (Fig. 5D).

### Increased LOXL4 expression in LOXL3-deficient lungs

**Figure 6:**
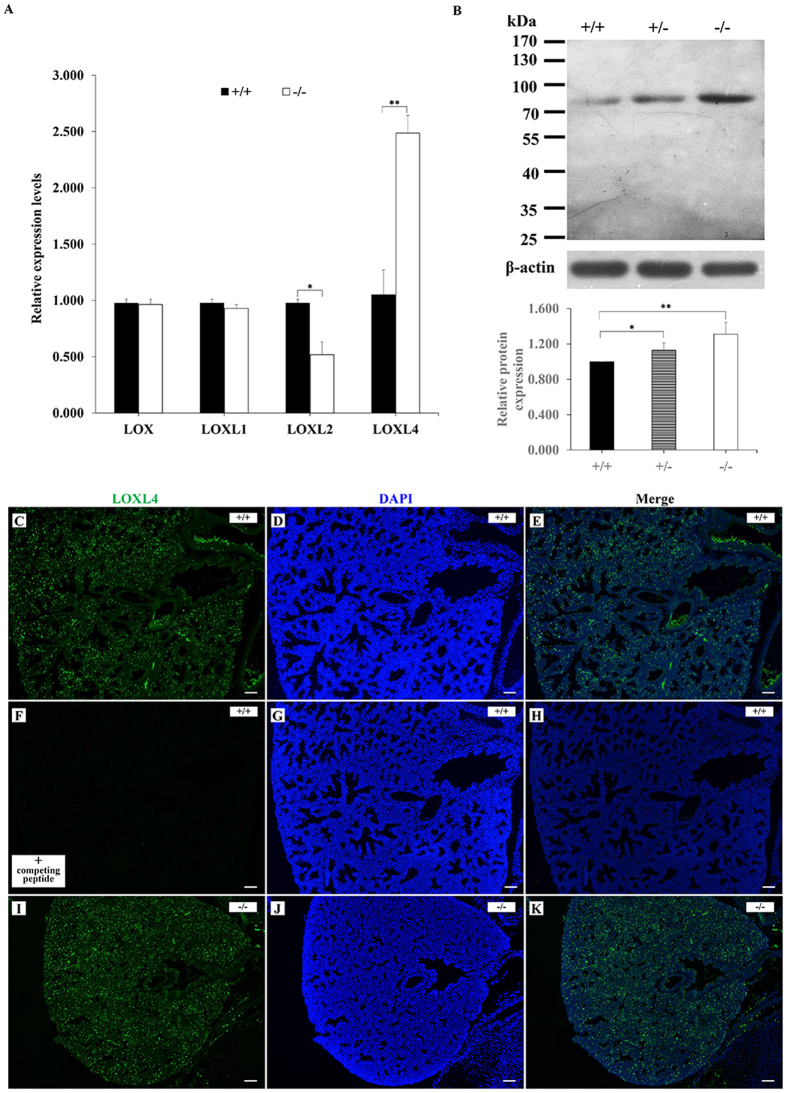
*Loxl4* expression in the lung. (**A**) The *Loxl2* mRNA level was significantly reduced in the *Loxl3*^−/−^ lungs compared with the *Loxl3*^+/+^ lungs. *P < 0.05. Conversely, the *Loxl4* mRNA levels in the *Loxl3*^−/−^ lungs were significantly increased compared with the *Loxl3*^+/+^ lungs. **P < 0.01. n = 3 in each group. (**B**) The LOXL4 protein levels in the lung were analysed by Western blot and normalized to β-actin expression. *Loxl3*^−/−^ lungs contained higher levels of the LOXL4 protein than *Loxl3*^+/+^ lungs. *P < 0.05, **P < 0.01. n = 5 in each group. (**C–K**) The distribution of LOXL4 in the *Loxl3*^+/+^ and *Loxl3*^−/−^ lungs was analysed by immunofluorescence. (**C–E**) LOXL4 was found to express widely in the *Loxl3*^+/+^ lung. (**F–H**) LOXL4 expression was not detected in the *Loxl3*^+/+^ lung in the presence of the LOXL4 competing peptide. (**I–K**) The high expression of LOXL4 was also observed in the *Loxl3*^−/−^ lungs. DAPI was used to stain the nucleus. Bar: 100 μm.

LOX family members are widely expressed in multiple tissues and many of these gene expression sites overlap^2^. We examined the expression of LOX family genes in mouse lungs by real-time PCR to investigate the effect of LOXL3 deficiency on other LOX family members. There was no significant difference in the *Lox* and *Loxl1* mRNA levels between the *Loxl3*^+/+^ and *Loxl3*^−/−^ mice (Fig. 6A). The *Loxl2* mRNA level was decreased in the *Loxl3*^−/−^ lungs (Fig. 6A). However, the *Loxl4* mRNA level was significantly increased in the *Loxl3*^−/−^ lungs compared with the *Loxl3*^+/+^ lungs (Fig. 6A). The Western blot results also indicated that the *Loxl3*^−/−^ lungs contained higher levels of the LOXL4 protein than the *Loxl3*^+/+^ lungs (Fig. 6B). We examined the distribution of the *Loxl4* gene in mouse lungs. Immunofluorescence staining showed that LOXL4 was widely expressed in *Loxl3*^+/+^ lungs (Fig. 6C–E). No fluorescence signal was detected in *Loxl3*^+/+^ lungs by the LOXL4 antibody in the presence of excess competing peptide (Fig. 6F–H). We also examined the high expression of LOXL4 in *Loxl3*^−/−^ lungs (Fig. 6I–K).

### Increased elastic fibres and desmosine/elastin ratios in HEK-293 cells transfected with the pENTER-C-GFP-LOXL4 vector

**Figure 7:**
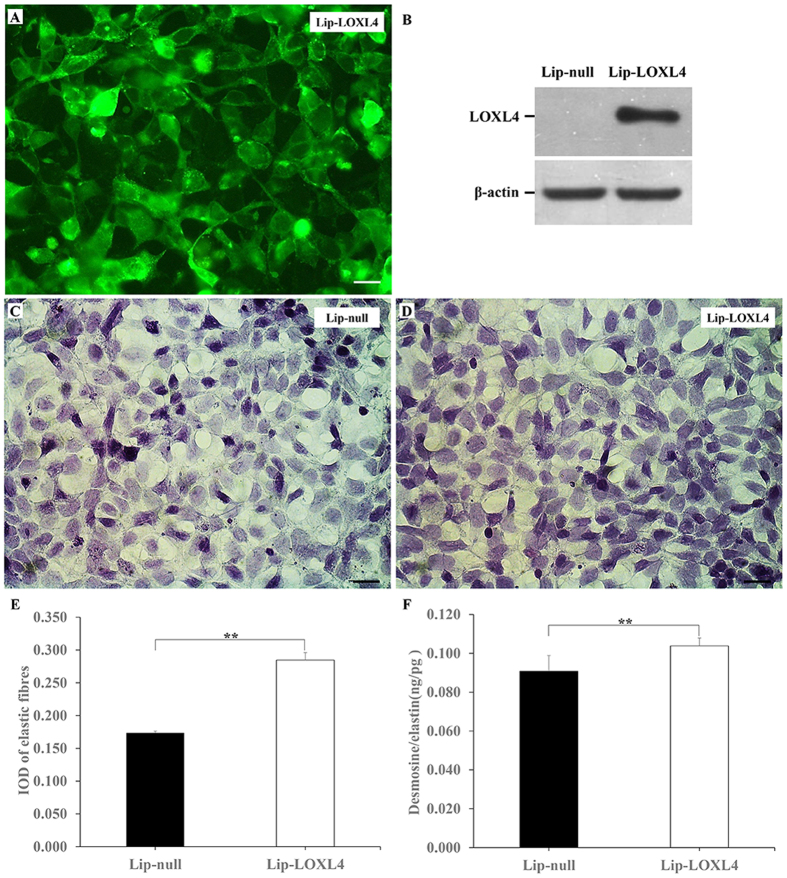
Changes in the elastic fibres and desmosine/elastin ratios in transfected HEK-293 cells. (**A**) GFP-positive signals for the LOXL4 protein were detected in transfected HEK-293 cells (Lip-LOXL4). Bar: 50 μm. (**B**) Western blots for LOXL4 and β-actin in Lip-LOXL4 cells and Lip-null cells were analysed. (**C,D**) Increased elastic fibres were observed in Lip-LOXL4 cells compared to Lip-null cells. Bar: 25 μm. (**E**) The mean IOD of the elastic fibres in the Lip-LOXL4 cells was significantly higher than in the IOD in the Lip-null cells. n = 4 in each group. **P < 0.01. (**F**) A higher desmosine/elastin ratio was also detected in the Lip-LOXL4 cells. n = 6 in each group. **P < 0.01.

Both excessive accumulation of elastic fibres and increased LOXL4 expression were observed in LOXL3-deficient lungs. An *in vitro* test was performed to investigate whether the excessive accumulation of elastic fibres was relevant to the increased LOXL4 expression. We constructed the recombinant pENTER-C-GFP-LOXL4 plasmid and transfected it into HEK-293 cells. GFP-positive signals for the LOXL4 protein were detected in the Lip-LOXL4 cells (Fig. 7A). LOXL4 was not expressed in the Lip-null cells, but LOXL4 was expressed at high levels in the Lip-LOXL4 cells (Fig. 7B). We found that Lip-LOXL4 cells exhibited a significant increase in elastic fibres compared with the Lip-null cells (Fig. 7C–E). We also examined the desmosine and elastin concentration in both Lip-LOXL4 cells and Lip-null cells. The desmosine/elastin ratio was significantly increased in the Lip-LOXL4 cells compared with the Lip-null cells (Fig. 7F).

### Expression of alveolar epithelial cell differentiation markers, tropoelastin, and transforming growth factor β1 (TGFβ1) in LOXL3-deficient lungs

**Figure 8:**
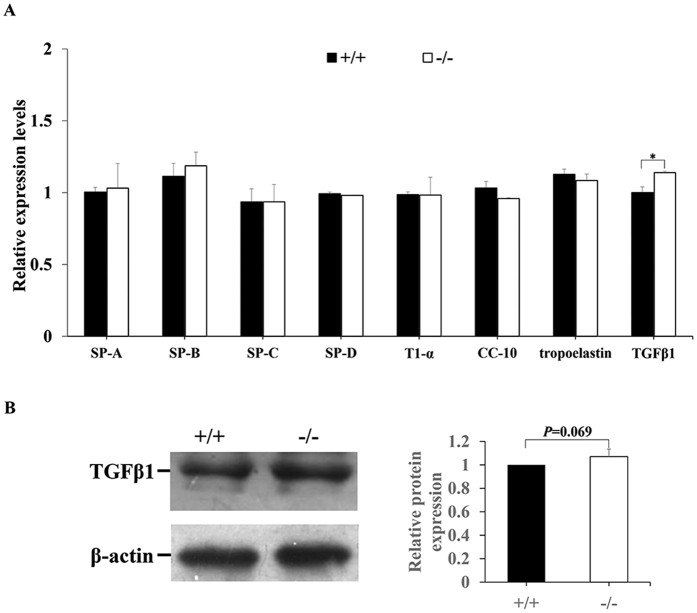
Changes in the alveolar epithelial cell differentiation markers tropoelastin and TGFβ1 in E18.5 *Loxl3*^−/−^ and *Loxl3*^+^/^+^ lungs. (**A**) There were no significant differences in the mRNA levels of *SP-A*, *SP-B*, *SP-C*, *SP-D*, *T1-α*, *CC-10* and tropoelastin between the *Loxl3*^−/−^ and *Loxl3*^+/+^ lungs. The *Loxl3*^−/−^ lungs exhibited a significant increase in the *TGFβ1* mRNA levels. n = 3 in each group. *P < 0.05. (**B**) Western blots for TGFβ1 and β-actin in E18.5 *Loxl3*^+/+^ and *Loxl3*^−/−^ lungs were analysed. The increased levels of the TGFβ1 protein were detected in the *Loxl3*^−/−^ lungs. n = 4 in each group. *P* = 0.069.

At a late stage of gestation, the foetal lung undergoes alveolarization, and peripheral alveolar epithelial cells differentiate into alveolar epithelial type I and type II cells. Alveolar epithelial type II cells produce surfactant proteins (SP-A, SP-B, SP-C, and SP-D), which are required for respiratory initiation or lung defence. T1-α is abundantly expressed in alveolar epithelial type I cells, and Clara cell protein10 (CC-10) is a non-ciliated, bronchial secretory cell marker. Our results showed a significant decrease in the size of the saccular spaces of the *Loxl3*^−/−^ lungs. We examined the expression of these marker genes in E18.5 mouse lungs. There was no significant difference in the mRNA levels of *SP-A*, *SP-B*, *SP-C*, *SP-D*, *T1-α*, and *CC-10* between the *Loxl3*^−/−^ and *Loxl3*^+/+^ lungs (Fig. 8A). The expression of the tropoelastin mRNA was similar in the *Loxl3*^−/−^ lungs and *Loxl3*^+/+^ lungs (Fig. 8A). Moreover, we also examined the expression of TGFβ1, which can induce LOXL4 expression^15^,^16^, in E18.5 mouse lungs. The *TGFβ1* mRNA levels were significantly increased in the *Loxl3*^−/−^ lungs compared with the *Loxl3*^+/+^ lungs (Fig. 8A). The level of the TGFβ1 protein was also increased in the *Loxl3*^−/−^ lungs compared with the *Loxl3*^+/+^ lungs, but the difference was not statistically significant (*P* = 0.069, Fig. 8B).

### Cell proliferation and apoptosis in the lungs of LOXL3-deficient mice

**Figure 9:**
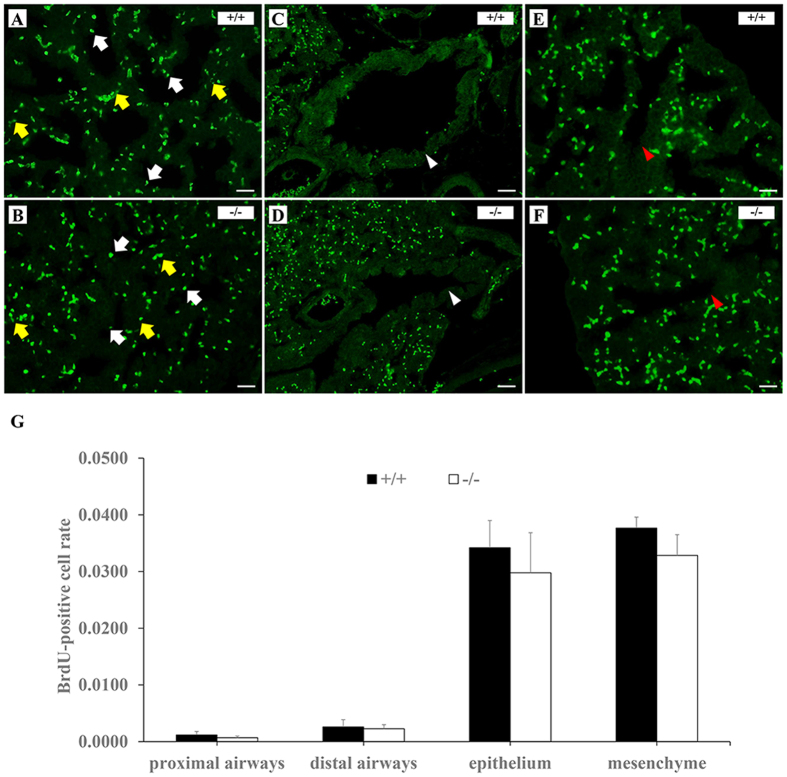
BrdU staining in the lungs from E18.5 LOXL3-deficient and wild-type mice. (**A–F**) Lung sections were stained for BrdU using fluorescently labelled antibodies. BrdU-positive cells (green) were primarily detected in the pulmonary epithelium (white arrows) and mesenchyme (yellow arrows) (**A,B**), and fewer BrdU-positive cells were detected in the proximal airways (**C,D**, white arrowheads) and distal airways (**E,F**, red arrowheads) of both the LOXL3-deficient and wild-type mice. (**G**) No significant differences were found in the number of BrdU-positive cells between the E18.5 LOXL3-deficient and wild-type lungs. n = 3 in each group. Bar: 25 μm.

**Figure 10:**
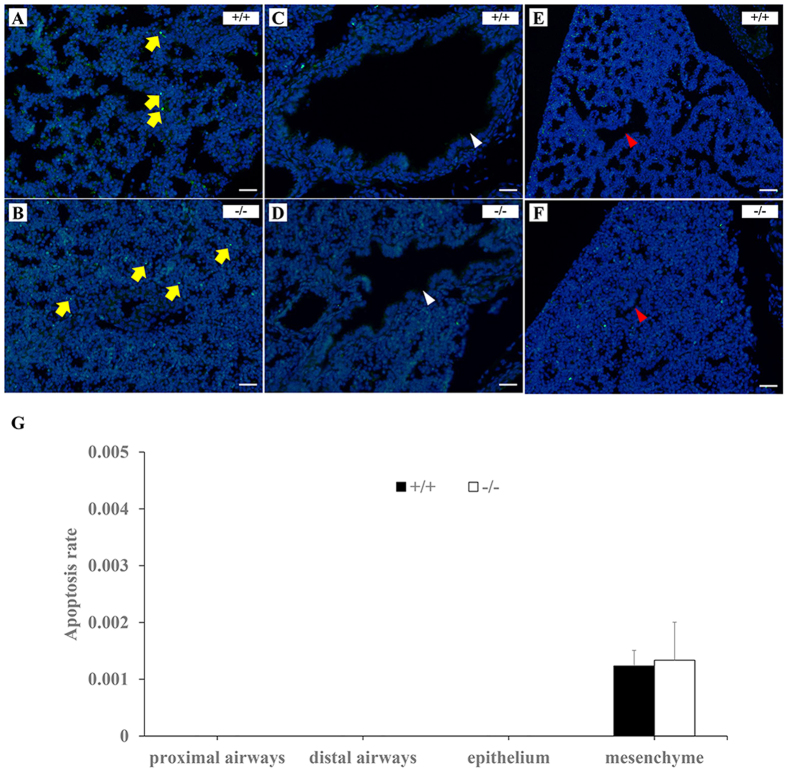
TUNEL staining in the lungs of E18.5 LOXL3-deficient and wild-type mice. (**A–F**) TUNEL-positive cells (green) were localized in the pulmonary mesenchyme (**A,B**, yellow arrows), and not in the proximal airways (**C,D**, white arrowheads), distal airways (**E,F**, red arrowheads), and epithelium of the LOXL3-deficient and wild-type mice. (**G**) The apoptosis rate in LOXL3-deficient lungs was not different from the wild-type lungs. DAPI was used to stain the nucleus. n = 3 in each group. Bar: 25 μm.

A significant abnormality in *Loxl3*^−/−^ lungs was identified, but it is uncertain whether the lack of LOXL3 affects cell proliferation and apoptosis in the lungs. We examined cell proliferation and apoptosis in the lungs. There was no significant difference in the number of BrdU-positive cells in the epithelium, mesenchyme, proximal airways, and distal airways between the *Loxl3*^−/−^ and *Loxl3*^+/+^ lungs (Fig. 9A–G). We did not observe apoptosis in the proximal airways, distal airways and epithelium of the LOXL3-deficient and wild-type mice. The apoptosis rate in the mesenchyme of the *Loxl3*^−/−^ lungs was similar to the apoptosis rate in the *Loxl3*^+/+^ lungs (Fig. 10A–G).

## Discussion

LOXL3 is similar to LOXL2 and LOXL4, and different from LOX or LOXL1 in structure^2^. *LOXL3*, a candidate human disease gene, was identified in a family with autosomal recessive Stickler syndrome^8^. In our previous study, we used a gene targeting strategy to generate LOXL3 knockout mice that exhibited severe craniofacial and spinal defects^13^. We also found that the saccular spaces in the mutant mouse lungs were smaller than those in the wild-type mouse lungs^13^. It was unclear whether the lack of LOXL3 affected embryonic lung development. Therefore, we investigated the effect of LOXL3 on embryonic lung development. In the current study, the most remarkable phenotype of the LOXL3-deficient mice was pulmonary hypoplasia at the end of the embryonic period. We found that the volumes and weights of the LOXL3-deficient mouse lungs were significantly decreased at E18.5. Paraffin sections and H&E staining showed a significant reduction in the saccular spaces of the LOXL3 knockout lungs in late pregnancy. Moreover, the significant decrease in MLI also reflected the decrease in the size of the saccular spaces in the LOXL3 knockout lungs. These results showed that the lack of LOXL3 disturbed normal lung development and induced pulmonary hypoplasia in LOXL3 knockout mice. We first revealed that LOXL3 was closely related to lung development. In our previous results, one characteristic of LOXL3-deficient mice was spinal abnormalities, specifically an abnormal backward bending of the vertebral column from E14.5 onwards^13^. The spinal deformity may result in changes in thorax dimension. In current study, we also examined the change in LOXL3-deficient mouse thoraxes during the embryonic period. We observed a significant decrease in the thoracic volume in the LOXL3-deficient mice. This result indicates that the lack of LOXL3 leads to a reduction in the thoracic volume. We are not sure whether the decrease in the thoracic volume was related to the abnormal lung development observed in the current study. However, in previous studies, restrictions of the thoracic volume impaired lung growth and led to pulmonary hypoplasia^17^,^18^. Thus, we suggested that the decreased thorax volume may have affected lung development.

We examined the expression of LOXL3 in the mouse lung to investigate the effect of LOXL3 on lung development. We discovered that mouse lungs express high levels of LOXL3. LOXL3 was predominantly expressed in the pulmonary mesenchyme. This result indicates that LOXL3 may affect the assembly of the extracellular matrix, such as collagen and elastin. In current study, we showed an increase in elastic fibres in LOXL3-deficient lungs caused by excessive elastin cross-links. In a previous study, newborn mice that were exposed to hyperoxia and treated with the lysyl oxidase inhibitor β-aminopropionitrile (BAPN) displayed an increased number of elastin cross-links^19^. Therefore, the increased elastic fibres in LOXL3-deficient lungs seem reasonable. In humans, the excessive accumulation of elastic fibres in the lungs was related to disorders such as bronchopulmonary dysplasia (BPD)^20^,^21^. An abnormal increase in elastic fibres has also been reported in different animal models of BPD. Increased elastin synthesis was found in the injured lungs of mouse pups with BPD^22^. The increased deposition of elastic fibres was associated with lung injury in premature lambs^23^. In addition, lung injury in rats was associated with altered elastin structure or distribution^24^. These previous studies indicated that the excessive accumulation of lung elastic fibres involved in the impaired lung development in many premature infants and animal models. Thus, we suggested that the increased accumulation of elastic fibres in the LOXL3-deficient lungs may be one of the factors causing pulmonary hypoplasia.

Lysyl oxidases are believed to initiate the formations of the lysine and hydroxylysine-derived crosslinks in collagens and lysine-derived crosslinks in elastin^1^,^3^. We examined the expression of other LOX family members. Reduced LOXL2 RNA expression was detected in LOXL3 knockout lungs. In a previous study^25^, the functions of LOXL2 were studied in both overexpression mice and knockout mice. Neither LOXL2 knockout mice nor LOXL2 overexpression mice displayed obvious lung defects, indicating that LOXL2 may not play an important role in lung development. Thus, we suggested that the decreased LOXL2 expression observed in the LOXL3 knockout was not sufficient to affect lung development. We also found that the LOXL4 content was significantly increased in LOXL3 knockout lungs. An *in vitro* assay was used to determine whether the excess elastin cross-links might be related to the increased LOXL4 expression. We constructed a recombinant LOXL4 eukaryotic expression plasmid and transfected it into HEK-293 cells. HEK-293 cells do not express LOXL4, but LOXL4 was expressed at high levels in the transfected HEK-293 cells. We detected an increase in elastic fibres and desmosine (elastin cross-links) as a result of the increased LOXL4 expression. Thus, we considered that the increased elastic fibres in the LOXL3-deficient lungs might be related to the high level of LOXL4. LOXL4 is regarded as a direct target gene up-regulated by TGFβ1^15^,^16^. Therefore, we also examined the expression of TGFβ1 expression. We detected an increase in TGFβ1 at the RNA level and an inconspicuous increase in TGFβ1 expression at the protein level. We suggested that TGFβ1 might be involved in the up-regulation of LOXL4 expression. However, it remains unknown how the lack of LOXL3 increases TGFβ1 expression, requiring future studies.

In previous studies, the deficiencies of many genes in the lung have been demonstrated to affect cell proliferation and apoptosis and disturb lung development^26^,^27^,^28^. We also examined cell proliferation and apoptosis in LOXL3-deficient mouse lungs. Normal cell proliferation and apoptosis were observed, suggesting that they do not appear to contribute to the defective phenotypes of the LOXL3-deficient lungs. In mammals, the lung is divided both structurally and functionally into two distinct components, the proximal airway that conducts air and the peripheral airway that mediates gas exchange^29^. At the terminal sac stage of lung development, peripheral alveolar epithelial cells differentiate into alveolar epithelial type I and type II cells, which express specific genes marking their cell identity and maturation. Alveolar epithelial type II cells produce surfactant proteins (SP-A, SP-B, SP-C, and SP-D), which are required for respiratory initiation or lung defence. The disrupted expression of SP-B and SP-C proteins could lead to severe respiratory distress in newborn infants^30^,^31^,^32^. T1-α is the alveolar epithelial type I cell differentiation marker, and CC-10 is a non-ciliated bronchial secretory cell marker^33^. We examined the expression of these alveolar epithelial cell differentiation markers in the lung, but no significant differences were detected. LOXL3 deficiency could not affect alveolar epithelial cell differentiation in the lung at a late stage of gestation.

In conclusion, LOXL3-deficient mice displayed pulmonary hypoplasia and deformed and smaller thoracic cavities. The excess elastic fibres observed in the LOXL3-deficient lungs might be related to increased LOXL4 expression. TGFβ1 might be involved in the up-regulation of LOXL4 expression in LOXL3-deficient lungs.

## Methods

### Animals

Heterozygous LOXL3-deficient mice (*Loxl3*^+/−^) were generated using a conditional gene targeting strategy (homologous recombination and Cre-recombination)^13^. Homozygous LOXL3-deficient mice (*Loxl3*^−/−^) were generated by intercrossing the *Loxl3*^+/−^ mice. All animal protocols were approved by the Ethics Committee of Shandong University. Animal management was performed strictly in accordance with the standards of the Animal Ethics Committee of Shandong University.

### Histological Analysis

E18.5 mouse foetuses were weighed, anaesthetized and euthanized. The lung tissues were removed and weighed. The lung weight/body weight (LW/BW) ratio was determined. For the transverse sections of the lungs and whole thoraxes stained with haematoxylin and eosin (H&E), Sirius red (cat. no. 43665, Sigma-Aldrich, USA) or resorcin-fuchsin (cat. no. G1054, Wuhan Goodbio Technology, CN), the embryos were isolated at different stages, fixed overnight in 10% neutral buffered formalin and embedded in paraffin. The deposition of elastic fibres in lung sections was estimated by resorcin-fuchsin staining. Computerized images were captured at a magnification of 100×. The deposition of elastic fibres was defined as the mean integral optical density (IOD) per lung section area. Elastic fibre-positive areas and the IOD values were analysed using Image ProPlus 6.0 software. Photomicrographs of Sirius red staining were analysed using Image ProPlus 6.0 software. The distribution of collagen fibres was calculated as the ratio of the IOD of the collagen fibres to the lung section area. For skeletal staining, skinned and eviscerated E18.5 foetuses were fixed overnight in 10% neutral buffered formalin. Before staining, the embryos were treated with 1% KOH for two days until they became clear. The bones were stained by incubating the embryos in a freshly prepared 0.1% Alizarin Red S (cat. no. A5533, Sigma-Aldrich, USA) for two days. The embryos were rinsed three times in 1% KOH (several hours each time). Finally, the stained bones were treated with a gradient of glycerol-KOH solutions.

### Morphometric analysis

The thoracic volume was measured in fixed, eviscerated, and weighed E18.5 foetuses. Liquid paraffin was poured into the thoracic cavity to the level of the diaphragm. The volume of the thoracic space was estimated from the weight and density of the paraffin casts^34^,^35^. Lung volume was also assessed as previously described^36^. The lungs were excised and their volumes were evaluated by fluid displacement^37^. Five sections from the same lobes of each lung sample were randomly chosen at approximately 250-μm intervals and stained with H&E. Images of the slides were digitally captured using SBI image 2.0 software version 2.0 (HK SAIBAO) with a line-counting tool. The number of distal air saccules across the terminal respiratory units was measured by radial alveolar counts (RAC) using a previously published method^38^,^39^. A perpendicular line was drawn from the centre of a respiratory bronchiole to the nearest connective tissue septum. The number of distal air saccules cut by this line was counted. At least 10 counts were performed in each lung sample. The mean linear intercept (MLI) was also estimated as previously described^40^,^41^,^42^. Briefly, the MLI was calculated as the linear sum of the lengths of all lines in all frames counted divided by the number of intercepts (defined as an alveolar septa intersecting with a counting line).

### Western blot and Immunofluorescence

E18.5 mouse foetuses were anaesthetized and euthanized. Lung tissues were incubated in cell lysis buffer (10 mM Tris, pH = 7.4, 1% Triton X-100, 150 mM NaCl, 1 mM EDTA, and 0.2 mM PMSF) and extracted using a homogenizer. The western blot protocol was performed using a previously described method^43^. The primary antibodies in this study used were rabbit anti-LOXL3 (1:100, cat. no. ESAP16939; Elabscience Biotechnology, CN), goat anti-LOXL4 (N-14) (1:200, cat. no. sc-48731; Santa Cruz Biotechnology, USA), and rabbit anti-TGFβ1 (1:500, cat. no. ENT4632; Elabscience Biotechnology, CN). The secondary antibodies in this study used were donkey anti-goat IgG-HRP (1:2000, cat. no. sc-2020; Santa Cruz Biotechnology, USA) and goat anti-rabbit IgG-HRP (1:5000, cat. no. ZB-2301; ZSGB-BIO, CN). The protein bands were quantified using NIH image analysis software (ImageJ Version 1.48V, National Institutes of Health, USA). Transverse sections of the foetal lungs were stained according to the H&E or immunofluorescence protocols. Immunofluorescence staining was performed using standard staining procedures with the following primary antibodies: rabbit anti-LOXL3 (1:50, cat. no. ESAP16939; Elabscience Biotechnology, CN), mouse anti-N-cadherin (1:200, cat. no. 66219-1; Proteintech Group, CN), rabbit anti-E-cadherin (1:500, cat. no. ENT1454; Elabscience Biotechnology, CN), and goat anti-LOXL4 (N-14) (1:200, cat. no. sc-48731; Santa Cruz Biotechnology, USA). The secondary antibodies consisted of rhodamine (TRITC)-conjugated AffiniPure goat anti-rabbit IgG (H + L) (1:200; cat. no. ZF-0316; ZSGB-BIO, CN), rhodamine (TRITC)-conjugated AffiniPure goat Anti-mouse IgG (H + L) (1:200; cat. no. ZF-0313; ZSGB-BIO, CN), fluorescein-conjugated AffiniPure goat anti-rabbit IgG (H + L) (1:200; cat. no. ZF-0311; ZSGB-BIO, CN) and fluorescein-conjugated AffiniPure rabbit anti-goat IgG (H + L) (1:200; cat. no. ZF-0314; ZSGB-BIO, CN). A competing peptide was used to block the antibody binding and test the specificity of the antibody. The competing peptides used in this study were the LOXL3 synthetic peptide (cat. no. ESAP16939-AN; Elabscience Biotechnology, CN) and LOXL4 peptide (cat. no. sc-48731p; Santa Cruz Biotechnology, USA).

### Real-time PCR

E18.5 mouse foetuses at were anaesthetized and dissected. The foetal lung tissues were analysed to determine the expression of LOX family genes, surfactant proteins, T1-α, CC-10 and the housekeeping gene mRNA using real-time PCR. The total RNA was extracted using TRIzol reagent (cat. no. 15596-026, Invitrogen, USA) according to the manufacturer’s instructions. The real-time PCR protocol was performed using a previously described method^44^. Primer sequences used for all real-time PCR experiments were also described previously^33^,^45^. The following primers were used: mLOX, forward (5′-TGCCAGTGGATTGATATTACAGATGT-3′) and reverse (5′-AGCGAATGTCACAGCGTACAA-3′); mLOXL1, forward (5′-AAGGCACAGCGGACTTTCTC-3′) and reverse (5′-GAACTCGTCCATGCTGTGGTAA-3′); mLOXL2, forward (5′-CAACCCCAAAGCCTATAAAACCT-3′) and reverse (5′-GCCCGTGCAGTTCATAGAAAA-3′); mLOXL4, forward (5′-TGGTGACCTGTCGGCAACT-3′) and reverse (5′-GAACTCCACTCATCACCACTTCTTT-3′); SP-A, forward (5′-GAGGAGCTTCAGACTGCACTC-3′) and reverse (5′-AGACTTTATCCCCCACTGACAG-3′); SP-B, forward (5′-GGCTGTGTCCCAGGTGTGCC-3′) and reverse (5′-AGGCTCCACAGCAGGGAGGG-3′); SP-C, forward (5′-TAGCCCCGAGTGAGCGAGCA-3′) and reverse (5′-GTGGGTGTGGAGGGCTTGGC-3′); SP-D, forward (5′-GCCTGGTCGTGATGGACGGG-3′) and reverse (5′-AGGGCCCTGCAACCCTGAGA-3′); T1-α, forward (5′-CAGGAGACGGCATGGTGCCC-3′) and reverse (5′-AGGCTTCGTCCCACGCTCTCT-3′); CC-10, forward (5′-GCGGGCACCCAGCTGAAGAG-3′) and reverse (5′-GAGCCGAGGAGACACAGGGCA-3′); tropoelastin, forward (5′-CTTTGGACTTTCTCCCATTTATCC-3′) and reverse (5′-GGTCCCCAGAAGATCACTTTCTC-3′); TGFβ1, forward (5′-TGACGTCACTGGAGTTGTACGG-3′) and reverse (5′-GGTTCATGTCATGGATGGTGC-3′); and β-actin, forward (5′-ACTATTGGCAACGAGCGGTT-3′) and reverse (5′-CAGGATTCCATACCCAAGAAGGA-3′).

### Hydroxyproline, Desmosine and Elastin Assay

E18.5 mouse foetuses were anaesthetized and dissected. The foetal lung tissue samples were used to analyse the amounts of hydroxyproline, desmosine and elastin. Hydroxyproline is a major component of collagen, and the hydroxyproline content accurately reflects the amount of collagen in the sample. Freeze-dried samples were hydrolysed and hydroxyproline was quantified using a hydroxyproline assay kit (cat. no. A030-2, Nanjing Jiancheng Bioengineering Institute, CN). Desmosine, a crosslinking amino acid in mature elastin, was quantified using an ELISA kit as previously described^46^. The mouse desmosine ELISA kit (cat. no. CSB-E14196m, Wuhan, CN) was purchased from Cusabio Biotech and performed according to the manufacturer’s instructions. Elastin was quantified using the mouse elastin ELISA kit (cat. no. CSB-EL007617MO, Wuhan, CN), which was purchased from Cusabio Biotech and performed according to the manufacturer’s instructions.

### Transfection of HEK-293 cells with eukaryotic recombinant plasmid

We obtained a LOXL4 (NM_032211) human ORF cDNA clone (cat. no. CH875610, ViGene Biosciences, CN); then we subcloned the LOXL4 cDNA fragments into pENTER-C-GFP. The recombinant expression plasmid pENTER-C-GFP-LOXL4 or blank vector pENTER-C-GFP was transfected into the HEK-293 cells using Lipofectamine 2000 (cat. no. 11668-019, Invitrogen, USA), according to the manufacturer’s instructions. Seventy-two hours after transfection, the number of GFP-positive cells was counted using a fluorescence microscope. HEK-293 cells that successfully expressed pENTER-C-GFP-LOXL4 were defined as the Lip-LOXL4 cells, and HEK-293 cells that successfully expressed pENTER-C-GFP were defined as the Lip-null cells. A portion of the transfected HEK-293 cells were washed with PBS, fixed with 4% paraformaldehyde, and then used to detect the deposition of elastic fibres by resorcin-fuchsin staining. The deposition of elastic fibres in cells was analysed using the same steps mentioned above. Other transfected HEK-293 cells were collected and used to detect the level of desmosine and elastin using the same steps mentioned above. The desmosine ELISA kit (cat. no. CSB-E12871h, Wuhan, CN) was purchased from Cusabio Biotech. The elastin ELISA kit (cat. no. CSB-E09338h, Wuhan, CN) was purchased from Cusabio Biotech.

### Cell Proliferation and Apoptosis Assay

At late gestation (E18.5), pregnant mice were injected with 100 μg BrdU/g body weight (cat. no. B5002, Sigma-Aldrich, USA) and euthanized. E18.5 mouse foetuses were anaesthetized and dissected. Transverse sections of the foetal lungs were treated as described in the H&E protocol. BrdU was detected according to the manufacturer’s instructions (Sigma-Aldrich). Apoptosis was examined via a TUNEL assay using an *in situ* cell death detection kit (cat. no. 12156792910, Roche, USA) with a modified protocol based on the manufacturer’s instructions.

### Statistical Analysis

The data are expressed as the means ± SD. Differences in the measured variables between the experimental and control groups were assessed using Student’s *t*-test. Differences were considered statistically significant at *P* < 0.05.

## Additional Information

**How to cite this article**: Zhang, J. *et al.* Loss of Lysyl Oxidase-like 3 Attenuates Embryonic Lung Development in Mice. *Sci. Rep.* **6**, 33856; doi: 10.1038/srep33856 (2016).
